# Online group psychodynamic psychotherapy—The effectiveness and role of attachment—The results of a short study

**DOI:** 10.3389/fpsyt.2022.798991

**Published:** 2022-07-28

**Authors:** Zbigniew Wajda, Agnieszka Kapinos-Gorczyca, Sebastian Lizińczyk, Katarzyna Sitnik-Warchulska, Bernadetta Izydorczyk

**Affiliations:** ^1^Faculty of Management and Social Communication, Institute of Applied Psychology, Jagiellonian University, Krakow, Poland; ^2^Mental Health Centre “Feniks,” Gliwice, Poland; ^3^Faculty of Psychology, SWPS University of Social Sciences and Humanities, Katowice, Poland; ^4^Faculty of Philosophy, Institute of Psychology, Jagiellonian University, Krakow, Poland

**Keywords:** online group psychotherapy, attachment style, group psychodynamic psychotherapy, effectiveness, internet-based intervention anxiety, online therapy, remote therapy, COVID-19

## Abstract

The role of remote treatment, including psychotherapy, has increased during the COVID-19 pandemic. The results of research in this area are promising, initially pointing to similar effectiveness for online psychotherapy as that of face-to-face psychotherapy. A significantly smaller amount of research has been conducted on online group psychotherapy, in particular, in the psychodynamic paradigm. Many authors have drawn attention to the need to conduct further research, considering specific patient features, for example, personality traits, attachment style, age, and other demographic variables. This study conducted pre- and post-treatment (10 weeks) and a 6-week follow-up, on the effectiveness of online synchronous group psychodynamic psychotherapy (via Zoom) taking into account patients’ attachment styles. Four main hypotheses were tested: H1: Patients will obtain a lower score in the attachment’s dimensions of anxiety and avoidance; H2: Patients will get a lower level of symptoms and sense of loneliness; H3: Patients will have increased self-esteem; and H4: The anxiety and avoidance dimensions of the attachment will be predictors for the effectiveness of online psychodynamic group psychotherapy. Twenty-two outpatients participated in the study, out of which 18 suffered from neurotic, stress-related, and somatoform disorders (F40-F48), and four suffered from a depressive episode (F32.0, F32.1) according to ICD-10. The results of the pre-treatment test showed a reduction in the global severity of psychiatric symptoms (*d* = −0.526) and depressive symptoms (*d* = −0.5), as well as an increase in self-esteem (*d* = 0.444) and feelings of loneliness (*d* = 0.46). A change in the attachment dimension, anxiety (*d* = −0.557) and avoidance (*d* = −0.526), was also observed. The above results were maintained in the follow-up test conducted after 6 weeks. Additionally, a reduction in the symptoms of social phobia was observed. Attachment dimensions were not a predictor of the effectiveness of psychotherapy, but a decrease in avoidance during therapy was a predictor of increased symptoms of pain. The results of the research are promising in terms of psychiatric symptoms and increased self-esteem. During therapy, there may be a favorable change in attachment dimensions, but this variable was not shown to be a predictor of results. These results suggest that more controlled research is required.

## Introduction

The COVID-19 pandemic introduced a number of new circumstances that impact patients’ mental health and treatment. The pandemic not only increased the fear of getting infected by the virus, and losing health and life, but also a number of fears regarding people’s functioning in society, such as isolation, losing a job, and so on. In many people, the pandemic caused symptoms of mental disorders, and in patients already undergoing treatment, stress and the severity of symptoms increased (1–3). Moreover, the preventive measure of social distancing has become an impediment to the provision of healthcare, patient consultation, and continuing therapy, including psychotherapy. Due to this, many psychiatrists, psychologists, and psychotherapists, at the beginning of the COVID-19 pandemic, decided to provide online counseling and therapy (4, 5). A survey conducted by the American Psychological Association (6), between April and May 2020, showed that 16% of clinicians were offering remote services in addition to providing in-person services, whereas, three-quarters (76%) of clinicians were solely providing remote services, primarily via phone, a designated platform, or through a videoconferencing software. One of the more popular forms of psychological help is psychotherapy, which in an online mode may take on various forms, such as self-administrated therapy, individual or group therapy that may make use of video calling, written chats, telephone calls, and so on. All these forms have been developing swiftly over recent years (7, 8).

This study considers group psychotherapy conducted through video communicators in real time. As per Weinberg and Rolnick (9), this term does not include help that can be offered via e-mail, chats (e-therapy), and computer programs for therapy, including virtual therapy. Individual psychotherapy is the standard form of psychotherapy, wherein the patient remains in direct individual contact with their therapist. The phenomenon of introducing group psychotherapy as a method of treatment for psychiatric disorders has been deemed a revolution in psychiatry (10). It is described as a “triple E treatment,” which means effective, equivalent, and efficient. Subsequent investigations have shown that group psychotherapy is effective, with its results being equivalent to individual psychotherapy, and at the same time, is far more effective than individual psychotherapy, both financially and in terms of managing psychotherapists’ time (11, 12). However, this does not mean that we know everything about group psychotherapy, especially online group psychotherapy sessions. The effectiveness of online individual psychotherapy has been confirmed (7, 13), while very little is known about online group psychotherapy. Therefore, online group psychotherapy is one of those areas that require further research. In the literature, we find several types of online therapeutic groups. Online groups are most often presented in the cognitive-behavioral (CBT) paradigm, using classic cognitive-behavioral interventions, including managing mood, increasing pleasant activities, managing negative thoughts, increasing positive thoughts, and planning for the future. Additionally, its use is seen in Dialectical Behavioral Therapy or Mindfulness-Based Stress Reduction training (14–16), which is also famously known as the third wave of CBT. In such groups, emphasis is placed on the active performance of tasks by participants, psychoeducation, and training in new skills. Other known forms of online group therapy are online support groups, which can be defined as “being together with people facing similar problems, sharing useful information on how to cope and solve those problems, and providing emotional support to each other” (17). Online group psychotherapy in the psychodynamic or interpersonal paradigm is described much less frequently in the literature. These approaches propose a smaller treatment program structure and a greater focus on spontaneous interactions among the participants, as well as between participants and group leaders. These interactions, along with the participants’ life stories, are then analyzed. According to Yalom and Leszcz (11), in such groups, a specific social microcosm is created, in which the participants’ intrapersonal and interpersonal problems of their everyday lives are played out during therapy. Psychodynamic and interpersonal approaches deal with a number of phenomena that are known to clinicians but are difficult to define in scientific terms, including group process, group development, transference and countertransference, group climate, group cohesion, group-as-a-whole, etc. The mechanisms of changing group members according to these approaches lie in close cooperation based on trust, even when conflicts and other difficult group situations arise. Group participants, together with group leaders, strive to make people aware of internal dynamisms and inadequate patterns of behavior that so far they had been unaware of. In addition, Yalom, based on his research (11), listed 12 non-specific therapeutic factors that contribute to participant change, hidden in group dynamics and individual member interactions. One of the most important factors in later studies turned out to be group cohesion, which is defined as the equivalent of a therapeutic relationship in individual therapy. Therefore, the psychodynamic and interpersonal approaches in group psychotherapy are based on the intense dynamics of interpersonal relationships (18, 19). The question of whether such a dynamic is possible when group psychotherapy takes place online, and whether this type of therapy is effective or not, still remains open. Weinberg (13) reviewed the literature and stated that only nine studies have been conducted on the effectiveness of online group psychotherapy; of which only one study concerned psychotherapy in the psychodynamic paradigm, while the others focused on CBT groups or support groups. In a study carried out by Lemma and Fonagy (20), patients with depression and anxiety (*n* = 8) participated in online Dynamic Interpersonal Therapy (DIT). No significant differences were discovered compared to the other two groups (self-help based on DIT, *n* = 8; non-specific mental well-being site, *n* = 8). However, the authors concluded that the reduction in symptoms appeared to be greater in the group with the therapist. Banbury et al. (21) reviewed the literature to determine the feasibility, acceptability, effectiveness, and implementation of health professional-led group videoconferencing to provide education or social support or both, in the home setting. Seventeen studies were systematically reviewed, and the results showed that support and educational online groups bring with them similar effects as face-to-face groups. The majority of these researches showed similar processes as in-person groups, such as group cohesion, and forging bonds with other participants. Improvement in the psychiatric health of the participants was also seen. However, the authors of the review indicate that further research is needed into the elements affecting effectiveness, as well as widening the results base on the subject of online groups, as the tests until now have been relatively few and varied in methodologies which have made it difficult to compare the results obtained. It may be said that online group therapy is a relatively new modality, still possessing too few items of research on its effectiveness. That said, the hitherto reports have been most promising. The majority of the trials in this area covered CBT groups, an extremely small number were concerned with psychodynamic and interpersonal online groups. Various studies have shown that in order to make the next step, research is needed, into the specific aspects of online group psychotherapy connected with the psychological features or traits of the patients as equally with the very specifics of this type of therapy (13, 22).

Some authors claim that online group psychotherapy can also bring benefits (3, 13, 23). In this era of the pandemic, when many people are isolating themselves from face-to-face contact with others, in accordance with instructions or out of fear of falling ill, they may experience a sense of loneliness. Brooks et al. (3) reviewed the effects of quarantine and found that there are long-lasting effects of quarantine that exist for years later. Holt-Lunstad et al. (24) have shown that together with a sense of loneliness and isolation increases the likelihood of death; while (23) argued that feelings of loneliness have long-term effects and that people may continue feeling lonely even when in the company of family or friends. Many authors argue that group psychology and support groups may constitute an effective form of countering loneliness and work on its effects (11, 17, 23, 25).

One of such important psychological features in the context of effectiveness and usefulness of online group psychotherapy may be the individual characteristics of patients’ attachment styles according to the attachment theory. The key assumption of attachment theory states that there is a biological tendency in humans to create close, emotional bonds with the caregiver whose function is to provide protection. During the early interactions of the child with their caregiver, the child creates specific mental structures, which in later life constitute patterns for creating relationships with others, ways of regulating emotions, and coping with problems (26). Empirical research has shown that these patterns continue into adulthood, and the characteristics of the attachment can be described in the two orthogonal dimensions: anxiety and avoidance. Anxiety is a dimension showing constant worry about whether the attachment figure will be available and sufficiently sensitive to the needs, while the avoidance dimension describes keeping distance in interpersonal relationships, which results from the fear of dependence, control, and rejection on the part of the other (27–29). Some patients present a high level of anxiety and avoidance in both dimensions, referred to as fearful or disorganized attachment style. They are compared by some researchers to disorganized infants because they do not be unable to establish an adaptive strategy in interpersonal relations. There is a parallel association between back-and-forth attachment of disorganized children in Strange Situations Procedure (28). Patients with such attachment characteristics, consequently in various studies, show the highest intensity of psychopathology. Although they can achieve positive results in psychotherapy, its course is most often difficult and sometimes can even lead to deterioration (30, 31). There exists an extensive subject literature using the concept of attachment in understanding psychopathology (30, 32), individual psychotherapy (33, 34) as well as family and couple therapy (35, 36). Until recently, there were relatively only a few publications showing the applicability of this theory in the phenomena within group psychotherapy, but in recent years interest in this topic has grown (23, 31, 37, 38). Until now, research conducted on in-person groups have shown that patients participating in group psychotherapy showing high anxiety and low avoidance in attachment relations had a growth therapeutic alliance with the group. This effect was not observed in groups with a behavioral-cognitive approach. The increase in therapeutic alliance turned out to have a significant meaning in obtaining good therapy outcomes in patients with binge eating disorders and for those displaying a high degree of anxiety in their attachment relations (39). Other researches, in large numbers of patients with various psychiatric diagnoses, have shown that the group climate acted as a moderator between the style of attachment and the effectiveness of psychotherapy (40). Tasca et al. (41, 42) argued that group psychotherapy could be particularly important for those who show a high level of anxiety in attachment relationships as it allows for new interpersonal relations to be experienced, which at the same time improves affect regulation. While Marmarosh and Tasca (43) suggested that probably the group could be a “safety base” model for patients, increasing group cohesion could subsequently be transferred to individual attachment traits. It is also important to note that the attachment style may constitute the predictor of the earlier termination of therapy for patients with a dismissive-avoidant attachment style. They more often give up on therapy before it has run its course. This happens because they are less inclined to reveal themselves in the group, the effects of therapy are less pronounced on them, and they also display a more negative attitude toward others in the group (39, 44). Flores (45), while explaining the mechanisms of change on the neurobiological level, argued that the therapeutic group, in a similar way to the therapist in individual psychotherapy, may establish a regulatory attachment relationship aimed at stabilizing physiology and emotions, and revising the emotional memory patterns. Fonagy and colleagues (46, 47) offered the term Epistemic Trust to explanations of changes during the psychotherapy course. Epistemic Trust describes the evolutionary ability to identify others as trustworthy. This ability is shaped by early interactions with the caregiver and modified throughout life by various life contexts, including psychotherapy. The authors of this concept argue that during the process of psychotherapy, three elements responsible for change may occur in particular: (a) sharing, which is about exchanging feelings and thoughts, and discussing internal experiences together, which leads to the second component: (b) “in-mode moments” where participants establish joint attention to explore and better understand one another’s emotional perspectives, and this, in turn, is related to (c) learnings, which is a process of acquiring new knowledge about social functioning and then applied it to other life situations (48). It can be hypothesized that these elements may have led to changes in “trust in others.” It should also be mentioned that the components leading to the change according to the Epistemic Trust concept are very similar to the therapeutic factors described in the literature mentioned by Yalom and Leszcz, e.g., imparting information, interpersonal learning, group cohesiveness (11).

To the best of our knowledge, the studies that have been conducted to date on attachment theory to understand online group psychotherapy did not consider the hypothesis that therapy via the Internet may be especially useful at the initial stage for attachment in patients (49). For such individuals, relations forged at a distance may feel to be less intrusive and as a result, they will be able to, albeit slower but with less discomfort, build bonds with others in the group. Taking into consideration the results discussed above and the literature gaps, we formed the following two research questions: (1) Is online group psychotherapy in the psychodynamic approach an effective form of therapy?; effectiveness is understood here as a lower score in the dimensions of attachment anxiety and avoidance, reduction of symptoms, reduced sense of loneliness, and increased self-esteem. In addition to reducing the level of individual symptoms, we selected two more indicators of effectiveness: (a) sense of loneliness as an important variable in the context of isolation related to the COVID-19 pandemic (23)—can online psychodynamic group psychotherapy reduce the sense of loneliness? (b) self-esteem—it is a variable very strongly related to mental health (50, 51), therefore, it is used very often in psychotherapy research, including group psychotherapy (52); second research question: (2) Can attachment’s dimensions anxiety and avoidance change in the course of online psychodynamic group psychotherapy and can this constitute the predictor for the effectiveness of such therapy? We hypothesized that after the online group psychotherapy in the psychodynamic approach:

H1: Patients will obtain a lower score in the attachment dimensions of anxiety and avoidance.

H2: Patients will have a lower level of symptoms and a sense of loneliness.

H3: Patients will have increased self-esteem.

H4: The anxiety and avoidance dimensions of the attachment will be a predictor for the effectiveness of online psychodynamic group psychotherapy.

## Materials and methods

### Participants

Twenty-two participants were included in this study: 13 women and 9 men, who were assigned to two therapeutic groups, one with 12 participants and the other with 10. Eighteen participants suffered from Neurotic, stress-related, and somatoform disorders (F40-F48), and four suffered from Depressive episodes (F32.0, F32.1), according to the ICD-10. In the beginning, the groups had 13 and 12 participants, respectively, but one person from the first group and two from the second group dropped out. The average age of the participants was 34 years (min = 21; max = 65). The characteristics of the research group are presented in Table 1.

**TABLE 1:** Selected characteristics of the study group (*n* = 22).

Variables	Study group *n* = 22	*n*
Sex	Men	9
	Women	13
Age	Mean	34
	Min-max	21–65
	Standard deviation	11.34
Education	Higher	14
	Secondary	8 (including six undergraduate students)
Marital status	Married	4
	bachelor/spinster	7
	partnership relationship	8
	divorced	3
Earlier participation in psychotherapy	Yes	17 (9 in individual, 6 in group)
	No	5
Psychiatric diagnosis according to ICD-10	Neurotic, stress-related and somatoform disorders (F40-F48)	18
	Depressive episode (F32.0, F32.1)	4
Psychiatric medicines taken in the course of psychotherapy	Yes	8
	No	14

### Procedure

The study was conducted from April to August 2021. Patients visiting the Center for the Treatment of Neurosis and Eating Disorders “Dabrowka” in Gliwice, Poland, as well as at several other treatment centers in the Silesian Voivodeship who received a diagnosis of neurotic, stress-related, and somatoform disorders (F40-F48), or mild/moderate depressive episode as per ICD-10 (F32.0, F32.1) and met the further criteria for inclusion or exclusion (described below) were offered the possibility of treatment in an online psychotherapy group. Patients who expressed a willingness to participate were contacted by the researchers and subsequently underwent two or three consultations with a clinical psychologist and a psychiatrist, during which they were tested for their suitability for group psychotherapy and conformity with the inclusion and exclusion criteria. The patients were prepared for psychotherapy and their relevant permission for participation was obtained. The patients were divided into two groups based on their preference for the days in question (Monday-Friday, Tuesday-Thursday), without randomization. The course of online group psychotherapy is described in the “Methods” section. Patients completed the research tools in an online form: 2 days before the beginning of therapy (pre-test), 2 days after the end of therapy (post-test), and 6 weeks after the completion of the online group psychotherapy (follow-up).

### Methods

#### Measures

The Experience in Close Relationships-Revised (ECR-R) (27, 53), in a short Polish version (54), was employed to test the dimensions and styles of attachment (1st and 10th week). This is a 16-item, Polish version of the instrument to test attachment in adulthood (54), which assesses attachment in close relationships based on two dimensions: anxiety and avoidance. The short version is based on the inclusion of items with the highest factorial load. The respondents expressed their opinion on statements regarding their functioning in close relationships on a 7-point scale from I (strongly disagree) to I (strongly agree). Cronbach’s alpha for anxiety was α = 0.89 and for avoidance α = 0.81.

The SCL-27PL (Symptom Checklist) is a test of neurotic symptoms. This is an abridged version of the SCL 90, which measures the general level of neurotic symptoms (Global Severity Index, α = 0.92) as well as separately measuring depressive symptoms (α = 0.87), vegetative symptoms (α = 0.77), symptoms of pain (α = 0.75), social phobia symptoms (α = 0.87), and agoraphobia symptoms (α = 0.85). This tool was developed by Hardt (55) and adapted in Polish by (56). The participants marked the intensity with which symptoms manifested themselves over the course of the previous week, on a 5-point scale (from never to extremely often). Questions 26 and 27 were removed from the tool as these concern symptoms of depression and suicidal thoughts and tendencies during the entirety of one’s life (these aspects were verified when recruiting individuals for group therapy in this study).

The SES—(Rosenberg Self-Esteem Scale) (57) is a 10-point scale to assess general self-esteem. The participants expressed their agreement with the statements on a 4-point scale. The Polish version presents good psychometric properties (7, 8), with Cronbach’s alpha α = 0.82 in adult groups (58, 59).

The De Jong Gierveld Loneliness Scale (60), adapted in Polish by (61), comprises 11 items related to both the emotional and social sense of loneliness. However, the Polish version is one-dimensional, testing only a generalized sense of loneliness (α = 0.89). The participants answered on a 5-point scale, from decisively *yes* to decisively *no*.

A questionnaire of our own with additional questions on demographic data and the course of prior treatment was also administered.

#### Patient qualification and preparation for therapy

Each of the patients interested in the project underwent a qualification in which they were assessed for their eligibility to participate in the online psychotherapy session, during a meeting with a clinical psychologist. In these meetings, basic patient data was taken along with a history of treatment. A medical diagnosis was made, and an interview presenting the fulfillment of the inclusion and exclusion criteria was conducted. The patients received information on the project and the principles in force in the therapeutic group both orally and in writing, and their questions were answered. The organization of the therapeutic group and the principles in force within it were modeled on the publications by Yalom and Leszcz (11) and Bernard et al. (12). The principles of online group work were modeled on the publication by Weinberg and Rolnick (8), and the principles of HIPAA were also taken into consideration.

The patients were included in the online psychotherapy group according to the following inclusion and exclusion criteria:

Inclusion criteria: (a) a patient during psychiatric testing was diagnosed with Neurotic, stress-related, and somatoform disorders (F40-F48) or mild/moderate Depressive episode (F32.0, F32.1), according to the ICD-10, and specialists saw indications for group psychotherapy treatment, (b) over 18 years of age (only those legally adult), (c) the agreement and motivation of the patient to both participate in the therapy and fulfill the requirements for completing the psychological questionnaires. Additionally, the patients had to meet the “technical criteria” for inclusion: (d) able to use a computer, navigate the Internet and cope with computer programs for remote communication, such as using emails, etc., (e) have an accessible working computer with a camera and Internet access, allowing smooth log-in to therapy as well as the absence of noticeable connection disturbances, (f) have headphones and a microphone, and (g) be able to allocate 2.5 h at a designated time for the therapy itself and available for a period of 10 weeks so that therapeutic sessions were not missed (ideally with full patient attendance at all session meetings), and (h) be able to isolate oneself from other members of the household during the therapeutic sessions in order to freely talk about important and intimate matters, with the simultaneous maintaining of the anonymity and dignity of other therapy participants.

Exclusion criteria: (a) diagnosis on a psychiatric examination of disorders other than neurotic, stress-related, and somatoform disorders (F40-F48), or mild/moderate Depressive episode (F32.0, F32.1) according to the ICD-10, (b) the absence of indications or counterindications for psychotherapy, (c) under 18 years of age (legal minors), (d) absence of consent or motivation to participate in the therapy or research program, (e) at present an extreme level of psychiatric symptoms, and (f) self-destructive thoughts and/or tendencies, suicidal thoughts and/or tendencies, a pronounced mental crisis which probably would require emergency medical and psychological intervention or hospitalization.

#### Course and organization of online group psychotherapy

Online group psychotherapy sessions took place during the afternoon hours, 2 times a week. Each meeting was divided into two therapeutic sessions—each session lasted 1 h, with a 15-min break between the sessions. The entire cycle lasted 10 weeks, that is, 40 therapeutic sessions in 20 meetings. Each group was led by an experienced group leader (a female psychologist) who had completed a full course of psychotherapy accredited by the Polish Psychiatric Association and had at least 2 years of experience in conducting therapeutic groups. The group leaders received the support of a psychotherapy supervisor for the duration of the online therapy.

##### Group process, group leader attitude, and therapeutic interventions

Psychotherapy was carried out within psychodynamic paradigm with elements of an interpersonal approach in accordance with the handbooks of Yalom and Leszcz (11), Bernard et al. (12) as well as Weinberg and Rolnick (8) in relation to the online group work. Psychotherapy was in adherence to good psychotherapeutic practices and incorporated the principles of the Ethics Code for Psychotherapists as approved by the Polish Psychiatric Association. One of the most important aspects of psychodynamic and interpersonal approaches is the focus on the group process. The term “process” refers to the nature of the relationship between the interacting individuals—the members and group leaders and must take into account many factors, including the inner psychological worlds of each member, interpersonal interactions, the strength of the group as a whole, the group’s clinical setting, and the wider socio-cultural environment. When talking about the process, we consider what the spoken words and the style of the participants reveal about their interpersonal relationships. Group leaders assess the metacommunication aspects of the message when the individual speaks in a certain way to a specific person at a certain time. Part of the message is delivered verbally and directly; part is expressed para-verbally (with nuance, inflection, pitch, and tone), and the rest is expressed behaviorally, even somatically, through posture and physical presence (11). So, the group participants were encouraged by the group leaders to interact spontaneously, talk about their problems, express their feelings, and in particular, analyze the causes of their problems and provide each other with feedback on their functioning. The main principles adopted by the group leaders were (a) avoidance of exposing oneself and directivity, and avoidance of giving advice and direct pointers; (b) repeated settings, being careful about any potential violating of group principles and the use of this material for therapeutic work; (c) accentuation of such work elements such as: group activeness in the analysis of one’s own problems, strengthening of group cohesion, encouraging a free exchange of feelings and interactions as well as feedback information on its functioning, employing the “here and now” technique, commenting on the group process, building a “group observing ego,” creating an atmosphere to experience corrective relations; (d) the interpretation of transference phenomena; (e) work on the defense mechanisms of patients by making them aware of the mechanisms; (f) strengthening the strength of the ego in individual patients; (g) analysis of the psychological functions of symptoms; (g) being aware patients’ interpersonal styles.

#### Compliance with ethical standards

The study was conducted in accordance with the Declaration of Helsinki, and the protocol was approved by the Bioethics Committee of the Jagiellonian University in Krakow (1072.6120.44.2021 of the 17th of March 2021). All participants were informed about its purpose and terms. Written informed consent was obtained from all participants.

#### Data analyses

As described above, three patients dropped out of the study and their data were not analyzed. The remaining data was complete as all the participants completed the questionnaires both before and after the psychotherapy cycle, and at the follow-up 6 weeks later. All data were complete, which results from the fact that the participants filled in the online versions of the questionnaires, in which the program did not allow them to bypass individual items. Statistical analysis was performed using Statistica 13.3 and Microsoft Excel (Microsoft Office 365), with a statistical significance of *p* < 0.5 adopted for the results. For the purpose of selecting appropriate statistical methods, the distribution of variables was verified as it met the conditions of a normal distribution. Due to multiple comparisons and proper determination of significance levels, Bonferroni correction for alpha levels was also used for all tested variables. Further data analysis was conducted in three basic steps. The first step was to establish the effectiveness of online group psychotherapy: at the beginning, basic calculations were made for the statistical description of the t-Student and of d-Cohen, and the Reliable Change Index (RCI) was also calculated. Reliable Change Index (RCI) is a statistic that can be used to find out whether a change in an individual’s score (for example, before and after some intervention) is statistically significant or not (based on how reliable the measure is). It is defined as the change in a client’s score divided by the standard error of the difference for the test(s) being used. We adopted a statistical significance of RCI ≥ 1.96 (62, 63). In the second step, the relationship between the attachment and the effectiveness of therapy was tested; with this aim in mind, the correlation between the attachment dimensions was checked, and in the two cases for which statistical significance was obtained regression analysis was performed. In the third step, an additional analysis of a clinical nature was conducted, which revealed the additional variables that were important in the course of therapy.

## Results

### Main results

Table 2 shows the average results together with the standard deviations for the individual variables, and for three time moments: immediately before the start of therapy, right after its completion, and 6 weeks after the end of the sessions (follow-up). Changes in all the variables were visible, particularly the differences between pre-test and post-test. Although for certain variables, for example, ECR-Anxiety, SCL-27: Global Severity Index, Depressive symptoms, and the Loneliness Scale further changes were visible after the completion of therapy itself.

**TABLE 2:** Pre-treatment, post-treatment and follow-up means and standard deviations (SD) of variables (*n* = 22).

		Pre-test 2-days before psychotherapy	Post-test 2-days after ending psychotherapy	Follow-up 6-weeks after psychotherapy
		Mean (SD)	Mean (SD)	Mean (SD)
ECR-R	Anxiety	32.318 (9.824)	26.409 (11.333)	23.591 (11.742)
	Avoidance	25.227 (8.847)	20.454 (8.733)	20.409 (8.661)
SCL-27PL	Global severity index	29.182 (15.534)	21.727 (12.837)	19.727 (14.102)
	Depressive symptoms	8.000 (4.909)	5.636 (4.686)	4.955 (4.952)
	Vegetative symptoms	4.227 (3.463)	3.455 (3.277)	2.864 (2.189)
	Agoraphobia symptoms	3.455 (4.469)	2.318 (3.483)	2.409 (3.912)
	Social phobia symptoms	7.818 (5.645)	5.909 (5.051)	5.091 (5.353)
	Symptoms of pain	5.682 (4.347)	4.409 (3.112)	4.409 (3.189)
SES	Global results	25.773 (7.104)	28.773 (6.361)	28.955 (7.088)
Loneliness scale	Global results	31.773 (8.275)	35.864 (9.433)	38.409 (9,226)

SD, Standard Deviation; ECR-R, Experience in Close Relationships-Revised; SCL-27PL, Symptom Checklist; SES, Self-Esteem Scale.

The subsequent stage in data analysis was the evaluation of the differences between the last and the first week of therapy (post-treatment—pre-treatment). Table 3 presents the differences that obtained a statistical deviation and what was the size/magnitude effect for the individual variables. At the level of the group, Anxiety (*t* = −2.83; *d* = −0.557) and Avoidance (*t* = −3.70; *d* = −0.546), and Global Severity Index (*t* = −2.57; *d* = −0.526). The depressive symptoms significantly decreased (*t* = −2.70; *d* = −0.500), and there was an increase in the level of self-esteem (*t* = 3.28; *d* = 0.460). The results obtained for the sense of loneliness were noteworthy as it rose significantly during therapy (*t* = 3.28; *d* = 0.460).

**TABLE 3:** Differences in the average results between post-treatment and pre-treatment, effect size and the t-Student test (*n* = 22).

		Differences in the average results between post-treatment and pre-treatment	d-Cohen	*T*	df	*p*
ECR-R	Anxiety	−5.9	−0.557	−2.83	21	0.010*
	Avoidance	−4.8	−0.546	−3.70	21	0.001*
SCL-27PL	Global severity index	−7.5	−0.526	−2.57	21	0.018*
	Depressive symptoms	−2.4	−0.500	−2.70	21	0.013*
	Vegetative symptoms	−0.8	−0.237	−1.03	21	0.315
	Agoraphobia symptoms	−1.1	−0.275	−1.84	21	0.080
	Social phobia symptoms	−1.9	−0.355	−1.75	21	0.094
	Symptoms of pain	−1.3	−0.344	−1.41	21	0.173
SES	Global results	3.000	0.444	3.282	21	0.004*
Loneliness scale	Global results	4.09	0.460	3.28	21	0.004*

*Statistically significant; ECR-R, Experience in Close Relationships-Revised; SCL-27PL, Symptom Checklist; SES, Self-Esteem Scale.

Further analysis was concentrated on whether the results were maintained 6 weeks after the end of therapy (follow-up). Table 4 shows a lack of statistically significant differences between the follow-up and post-treatment, which may be interpreted as the maintaining of results 6 weeks after the end of therapy. It follows to take note of the social phobia symptoms, which did not show a statistically significant difference immediately after the end of therapy (*t* = −1.75; *p* = 0.094, Table 3) but 6 weeks later did (*t* = −2.52; *p* = 0.020, Table 4).

**TABLE 4:** Difference in the average results between follow-up, pre-treatment and post-treatment and the t-Student test (*n* = 22).

		Follow-up and pre-treatment				Follow-up and post-treatment			
		Difference of averages	*t*	df	*p*	Difference of averages	*t*	df	*p*
ECR-R	Anxiety	−8.7	−4.37	21	0.001*	−2.82	−1.44	21	0.166
	Avoidance	−4.8	−3.03	21	0.006*	−0.05	−0.03	21	0.975
SCL-27PL	Global severity index	−9.5	−3.00	21	0.007*	−2.0	−0.59	21	0.565
	Depressive symptoms	−3.0	−2.36	21	0.028*	−0.7	−0.64	21	0.527
	Vegetative symptoms	−1.4	−1.67	21	0.110	−0.6	−0.8	21	0.430
	Agoraphobia symptoms	−1.0	−1.32	21	0.202	0.1	0.14	21	0.893
	Social phobia symptoms	−2.7	−2.52	21	0.020*	−0.8	−0.59	21	0.560
	Symptoms of pain	−1.3	−1.29	21	0.212	0.0	0.00	21	1.000
SES	Global results	3.182	2.138	21	0.041*	0.182	0.144	21	0.887
Loneliness scale	Global results	6.636	3.841	21	0.001*	2.545	1.340	21	0.192

*Statistically significant; ECR-R, Experience in Close Relationships-Revised; SCL-27PL, Symptom Checklist; SES, Self-Esteem Scale.

In the next step, the Reliable Change Index (RCI) was employed to show what number of patients who experienced improvement or deterioration (a statistical significance of RCI ≥ 1.96 was obtained). The largest number of people showed improvement in self-esteem (9; 40.9%), Global Severity Index in SCL-27 (6; 27.3%), Depressive symptoms (7; 31.8%), and Social phobia symptoms (6; 27.3%). Deterioration was noted in the largest number of individuals with Vegetative symptoms (7; 31.8%). All the results are presented in Table 5.

**TABLE 5:** Number and percentage of patients who experienced reliable change (post-treatment—pre-treatment) (*n* = 22).

		Reliable Change Index for post-treatment-pre-treatment		
		Improved	Unimproved	Deteriorate
SCL-27PL	Global severity index	6 (27.3%)	15 (68.2%)	1 (4.5%)
	Depressive symptoms	7 (31.8%)	15 (68.2%)	0 (0%)
	Vegetative symptoms	4 (18.2%)	11 (50.0%)	7 (31.8%)
	Agoraphobia symptoms	3 (13.6%)	18 (81.8%)	1 (4.5%)
	Social phobia symptoms	6 (27.3%)	15 (68.2%)	1 (4.5%)
	Symptoms of pain	2 (9.2%)	20 (90.9%)	1 (4.5%)
SES	Global results	9 (50%)	11 (50.0%)	2 (9.2%)
Loneliness scale	Global results	1 (4.5%)	17 (77.3%)	4 (18.2%)

SCL-27PL, Symptom Checklist; SES, Self-Esteem Scale.

At subsequent stages, statistical analyses were to answer the question of whether the attachment dimensions and their change could constitute a predictor of the effectiveness of online group psychodynamic psychotherapy. With this goal in mind, the first step was made in establishing the r-Pearson correlation between the attachment dimensions before therapy and the effects of therapy after its completion. As Table 6 shows, no statistically significant correlations were observed. Subsequent analysis was conducted on correlations between the results showing a change in the attachment dimensions (the difference of the averages at ECR-R between post-treatment and pre-treatment). Two statistically significant correlations were observed: the change in the attachment anxiety dimension was positively correlated with the social phobia symptoms (*r* = 0.444), while the avoidance dimension negatively correlated with the symptoms of pain (*r* = −0.501) in SCL-27PL. The remaining results were not statistically significant. The results are presented in Table 7.

**TABLE 6:** Correlations between attachment dimensions before therapy and the change in the severity of symptoms, self-esteem and the sense of loneliness (*n* = 22).

		ECR-R pre-treatment	
Difference in the averages between post-treatment and pre-treatment		Anxiety	Avoidance
SCL-27PL	Global severity index	−0.095	0.381
	Depressive symptoms	−0.190	0.173
	Vegetative symptoms	−0.038	0.343
	Agoraphobia symptoms	0.109	0.337
	Social phobia symptoms	−0.071	0.385
	Symptoms of pain	−0.080	0.077
SES	Global results	0.391	−0.192
Loneliness scale	Global results	−0.282	−0.388

ECR-R, Experience in Close Relationships-Revised; SCL-27PL, Symptom Checklist; SES, Self-Esteem Scale.

**TABLE 7:** Correlation between the change in the attachment dimensions over the course of therapy and the change in the severity of symptoms, self-esteem and the sense of loneliness (*n* = 22).

		Changes in ECR dimensions	
Difference in the averages between post-treatment and pre-treatment		Anxiety	Avoidance
SCL-27PL	Global severity index	0.401	−0.282
	Depressive symptoms	0.327	−0.0067
	Vegetative symptoms	0.118	−0.313
	Agoraphobia symptoms	0.190	−0.155
	Social phobia symptoms	0.444*	−0.085
	Symptoms of pain	0.207	−0.501*
SES	Global results	0.401	−0.282
Loneliness scale	Global results	0.327	0.067

*Statistically significant; ECR-R, Experience in Close Relationships-Revised; SCL-27PL, Symptom Checklist; SES, Self-Esteem Scale.

At the subsequent stage, an analysis of the regression of variables that had obtained a statistically significant correlation was conducted. The model for anxiety dimension and social phobia symptoms turned out to be statistically insignificant [Adjusted *R*^2^ = 0.155; *F*_(2, 19)_ = 2.93; *p* < 0.07]. Whereas, the change in avoidance attachment over the course of therapy turned out to be a significant predictor of symptoms of pain [Adjusted *R*^2^ = 0.297; *F*_(2, 19)_ = 5.44; *p* < 0.01]. A detailed analysis of regression is shown in Table 8.

**TABLE 8:** The results of regression analysis for anxiety and avoidance dimensions (ECR-R) and social phobia symptoms and symptoms of pain (SCL-27PL) (*n* = 22).

Independent variables (Measured by ECR_R)	Dependent variables (Measured by SCL-27PL)	Standardized factors β	Adjusted *R*^2^ F—statistics	Summary
Anxiety	Social phobia symptoms	0.493	Adjusted *R*^2^ = 0.155; *F*_(2, 19)_ = 2.93; *p* < 0.07	Irrelevant model
Avoidance	Symptoms of pain	−0.584	Adjusted *R*^2^ = 0.297; *F*_(2, 19)_ = 5.44; *p* < 0.01	Relevant model

### Additional analyses

At the final stage, additional analyses were conducted which could be especially useful in the clinical context. These analyses were conducted for selected variables: attachment (ECR-R), Global Severity Index (SCL-27PL), self-esteem (SES), and the sense of loneliness (Loneliness Scale).

By means of medians, the results of attachment dimensions were divided: anxiety and avoidance from the pre-therapy data were divided into 4 attachment styles for the individual participants. Table 9 presents the division of the 25 participants into particular attachment styles (together with those participants who dropped out, as well as the number and percentage of participants with a specified attachment style that obtained a significant improvement or deterioration in the selected variables on the basis of the Reliable Change Index (RCI).

**TABLE 9:** The attachment style of participants and the improvement or deterioration during the course of therapy in selected variables.

		Attachment styles			
		Secure (Low anxiety, low avoidant)	Preoccupied (High anxiety, low avoidant)	Dismissing-avoidant (Low anxiety, high avoidant)	Fearful (disorganized) (High anxiety, high avoidant)
Participants (*n* = 25)		7 (28%)	6 (24%)	5 (20%)	7 (28%)
Drop out (*n* = 3)		1 (4%)	1 (4%)	0 (0%)	1 (4%)
Global severity index (*n* = 22)	Improved	4 (18.2%)	1 (4.5%)	0 (0%)	1 (4.5%)
	Deteriorate	0 (0%)	0 (0%)	1 (4.5%)	0 (0%)
Self-esteem SES (*n* = 22)	Improved	2 (9%)	4 (18.2%)	1 (4.5%)	4 (18.2%)
	Deteriorate	0 (0%)	0 (0%)	1 (4.5%)	1 (4.5%)
Loneliness scale (*n* = 22)	Improved	0 (0%)	0 (0%)	0 (0%)	1 (4.5%)
	Deteriorate	1 (4.5%)	0 (0%)	2 (9%)	1 (4.5%)

In the first part of Table 9, the 25 people who qualified for the study were divided by individual attachment styles. The analysis showed that among participants who dropped out of group psychotherapy, each presented a different attachment style (secure, preoccupied, and fearful attachment style). The rest of Table 9 shows that a decrease in symptoms (Global Severity Index) was achieved by most people with a secure attachment style (4, 18.2%), while the increase in self-esteem was seen in participants with a preoccupied attachment style and fearful attachment style (both 4, 18.2%). Only one person experienced less loneliness (4-5%, with a fearful attachment style).

## Discussion

Online group psychotherapy is a relatively new modality that has been covered by only a limited amount of research into its effectiveness, especially within the psychodynamic paradigm. There are also shortfalls on the subject of patient traits connected with the course and effect of therapy. The presented research included 22 participants assigned to two psychotherapeutic groups conducted online (via videoconferencing) in the psychodynamic paradigm. It was tested whether such therapy could reduce patients’ symptoms, increase their self-esteem, and decrease their sense of loneliness. We especially investigated whether the patient’s attachment styles could change during the psychotherapy course and whether it could be a predictor of changes in symptoms, self-esteem, and sense of loneliness in such type of psychotherapy.

### Online group psychotherapy and attachment changes

To begin with, we were concerned with whether online psychodynamic group psychotherapy could be an effective form of treatment for patients who suffer from neurotic and depressive disorders. The first hypothesis was that patients would obtain a lower score in the attachment dimensions, that is, anxiety and avoidance. Previous research (64–66) showed that changes in attachment characteristics may be possible during group psychotherapy, especially based on interpersonal relationships but it was never tested in online group psychotherapy. Therefore, to the best of our knowledge, this is the first research project that examines these aspects in group psychotherapy conducted online. In our study, the average levels of anxiety and avoidance dimensions were reduced, and the effect size for the entire group was moderate for both of these dimensions. In the 6 week follow-up study after psychotherapy, these results were maintained, but there were no further statistically significant changes. We can therefore conclude that the first hypothesis was confirmed. Such results are somewhat surprising, considering the fact that the relationships of the group participants were online only. The attachment style as a research variable shows relatively high stability over time, and the online psychotherapy itself was short (only 10 weeks). Skentzos et al. (67) indicated that the group may be understood as a social microcosm that provides members with corrective emotional experiences. The authors also emphasized that the therapeutic effect is mediated by group cohesion and can also be moderated by the attachment style of the psychotherapist (68).

### Mental symptoms, sense of loneliness, and self-esteem

The second hypothesis was that the patients will get a lower level of symptoms and a sense of loneliness. In the first part of this hypothesis, the results obtained were fairly promising because the average global severity of symptoms tested had significantly reduced at the level of the whole group, and the effect size was moderate. An equal significant reduction was obtained in depressive symptoms. The remaining domains in the post-test did not obtain a statistical significance, although in all these domains the average result was reduced. Interesting results were also obtained in the follow-up testing where the majority of results maintained a further downward trend, and the effects for the global severity index and depressive symptoms were maintained at the level of statistical significance. However, the most interesting result was obtained with regard to social phobia symptoms where the result was not statistically significant directly after the end of therapy, but 6 weeks later it was. One may generally say that the results obtained are compared to those obtained for in-person group psychotherapy for anxiety disorder and depression, where for the majority of these, the effect size takes on a value from average to high (52, 69–71). As mentioned in the introduction, so far there has been only one study on online group psychotherapy in the psychodynamic paradigm (20), where there were no significant differences between online psychodynamic groups, self-help groups, and participants using a mental well-being site. Therefore, the results obtained in our research can be compared to online groups conducted in the CBT paradigm or online support groups. For example, (72) obtained the effect size of *d* = 0.39 for the online CBT paradigm in overall mental health, *d* = 0.77 for depression, and *d* = 0.74 for anxiety. Much lower results were obtained in the study by Breuer and Barker (17) where the patients (*n* = 9) participated in a 10-week online support group, obtaining improvements in depression symptoms (*d* = 0.33) and depression self-stigma (*d* = 0.33). However, no change was obtained in the remaining aspects including the sense of social support.

Many pieces of research related to group psychotherapy or other group online interventions were conducted within the cognitive-behavioral paradigm, with the use of relaxation techniques or mindfulness, and also in groups of somatic patients (for example, with a diagnosis of cancer or HIV), through the use of various statistical analyzes (22). Therefore, it is extremely difficult to compare the results of our research with those accessible in the subject literature for online group psychotherapy.

The second part of this hypothesis concerns the levels of sense of loneliness which during the COVID-19 pandemic has become a very important aspect. A negative result was observed, that is, the sense of loneliness grew during the course of therapy, and this trend was maintained till 6 weeks after its completion. Although the results obtained point to a sense of loneliness in patients over the course of group online psychotherapy, they did not hamper the improvements in the global severity of psychiatric symptoms, depressive symptoms, social phobia symptoms, or self-esteem. This is puzzling as it is well-known that being socially connected, positively influences psychological and emotional wellbeing, physical health, etc. (73). At the same time, it is noteworthy that the existing subject literature mentions that online psychotherapy may introduce or intensify a sense of loneliness [e.g., (8–74)]. However, these hypotheses did not lead to any appropriate empirical confirmation on the basis of psychotherapy, and in particular that of group psychotherapy. The evaluation of psychological interventions using this same instrument, as in the present research (De Jong Giervald Loneliness Scale), has also been carried out by Kahlon et al. (75) who obtained a reduction in the sense of loneliness over the course of 4 weeks (effect size *d* = 0.17). However, one cannot directly compare these results with ours because the research used individual, telephonic, and daily intervention over a period of 4 weeks and their therapeutic procedures were significantly different from ours. Some of the last tests showed that the relationship between a sense of loneliness and problems with psychological health is moderated by a variety of factors such as employment, work relations, life conditions, a history of medical problems, or directly a fear expressed over COVID-19 (76).

Summarizing the results of the second hypothesis, it should be stated that online group psychotherapy may be effective in reducing psychiatric symptoms, but at the same time, it does not change or even increase the participants’ sense of loneliness. The aforementioned results constitute an important source of information in a clinical context, that is, for patients for whom the main problem is a sense of loneliness such a type of treatment would possibly be unbeneficial. It is also important to remember that such a result could have many causes and this aspect requires further research, particularly with regard to a comparative group working in person.

We obtained results that self-esteem increased significantly during the course of treatment while the effect was equally maintained 6 weeks after the end of therapy. Therefore, it may be hypothesized that the changes, that is, an increase in the domain of self-esteem could have been connected with changes in social phobia symptoms, and on the basis of feedback. Self-esteem is an extremely common variable tested in the context of psychotherapy. Grah et al. (77) confirmed that long-term psychodynamic group psychotherapy improved wellbeing, including self-esteem in family members of persons with psychotic disorders. An increase in self-esteem is equally related to a person’s positive functioning and better psychological health (50, 78–80). Therefore, the result obtained should be deemed beneficial for patients (31, 60–62).

### Attachment style and effectiveness of online psychodynamic group psychotherapy

The present research has shown that attachment and its change during the course of psychotherapy may be the predictor, of treatment outcomes, therefore the second research question concerned specifically this very aspect. A dependence between pre-treatment attachment and outcomes was not discovered, yet a positive correlation was obtained between the anxiety dimension and the global severity index, which means that together with the change in the anxiety dimension there is a proportionate change in the general level of symptoms. However, as the subsequent analysis using regression analysis did not confirm the predictive role of anxiety dimension change in relation to the global severity index, the model turned out to be irrelevant. Furthermore, the revealed negative correlation between the change in avoidance dimension and symptoms of pain, and the regression analysis showed that the change in avoidance dimension is a significant predictor of increased symptoms of pain in the course of therapy. This result is puzzling but at the same time very interesting. We are unaware of any research in which similar results have been obtained. The results of various researches beyond the domain of psychotherapy show that insecure attachment correlates with a strong sense of pain, but results from the particular dimensions of anxiety vs. avoidance are ambiguous and indicate the connection of the anxiety dimension with a stronger experience of pain (81). Many authors point to the correlation between the experience of trauma, attachment styles, and somatization (82, 83) but equally to the relationship between mentalization deficits and the misinterpretation of bodily states, as patients with mentalization deficits may confuse emotional states and physical sensations which constitute an important element in the understanding of the psychosocial factors for the explanation of any pain experienced (84). Unfortunately, these aspects were not explored in our research, consequently, none of these may be either confirmed or rejected. According to attachment theory, avoidant patients will use a deactivating strategy to cope with emotions which are based on reducing their attachment needs and generally the conduct of “not feeling” equally in the case of the body, which Fosha (85) has deemed as “dealing but not feeling.” Possibly this could constitute an explanation for the results obtained in the research, that is, during the reduction of an avoidant form of behavior combined with the changing mechanisms of deactivation, patients are in a state to feel more, including the symptoms generated by the body, which may be perceived as a worsening of wellbeing in the course of therapy. A separate area for analysis could be the specifics of online group psychotherapy, where the body is not directly involved and patients do not see themselves as a whole; they equally do not exchange physical gestures such as shaking hands, embracing/hugging, etc., which could have a significance in the detection of bodily signals. More research into these matters is required to answer the question of whether this is a characteristic of group online therapy or whether it exists equally in in-person therapy.

The presented research has not confirmed the hypothesis (49) that online psychotherapy may be particularly useful at the initial stage of treatment for avoidant patients, that is, for those who experience discomfort from being in intimate relationships.

Beyond hypothesis evaluation, we included additional results with the division of patients into four attachment styles. It can be seen here that there is no dominant subgroup among participants who dropped out of therapy (some studies have indicated that people with high avoidance will most often drop out of in-person psychotherapy groups). Moreover, people with fearful/disorganized attachment styles (high on both anxiety and avoidance dimensions were also able to use online group psychotherapy, e.g., four participants with this attachment style significantly increased their self-esteem). This is important because, as noted in the Introduction, this group of patients has difficulties using psychotherapy (30, 86). However, it should be noted that patients with low or moderate severity of psychopathology were carefully selected for the project—so these results will require further research, taking into account a wider group of patients, including those with more severe psychopathology.

It is important to note that attachment was tested by means of a questionnaire and it would be interesting to employ the Adult Attachment Interview to test this particular aspect. Additionally, attention should be drawn to the fact that research into group psychotherapy conducted in-person in groups has shown how avoidant patients more often drop out (38). While in contrast, (87) have found that the positive association between the therapy group’s and the individual’s report of group atmosphere was significant only for those with higher attachment avoidance. Earlier, Dozier and Kobak (88) had made it known that despite withdrawal behavior, patients with avoidant attachment still may be emotionally or physiologically triggered in the context of an interpersonal group. The entirety of the subject literature displays a huge discrepancy in this area, while (40) suggest that the differences in the results may be explained through differences in the operationalization of variables (various conceptions and instruments employed to measure attachment), the individual psychological differences of patients as well as the differences in relation to treatment, such as the composition of the therapy group, the gender of participants and even the traits displayed by the group leaders. Our research has introduced at least one significant additional variable, namely the notion of remote treatment (via the Internet). Marmarosh et al. (31) add that it follows to take into consideration the different mediating variables, which could be particularly important for a patient displaying a different attachment style, for example, the level of a therapist’s acceptance and the availability for avoidant patients, or the group climate for preoccupied group members (those high on the anxiety dimension).

### Limitations

The results presented in the current article are drawn from a short research program, that aimed to generate initial data, yet the research has its limitations. The most important of which are the small number of participants, the lack of comparative and control groups as well as the absence of randomization. Moreover, short self-evaluating/descriptive scales were employed, limiting the objectifying indicators of change. The use of questionnaires, including to assess attachment dimensions, is also a limitation of our research. On the one hand, the ECR-R is often used and recommended (89), also in clinical practice, for a quick diagnosis of the patient’s functioning style, but the Adult Attachment Interview is considered the best tool in this respect. AAI presents the best psychometric properties and is recognized as the gold standard in research; AAI remains the most established instrument, with excellent psychometric properties (90).

Another important limitation is the lack of measurement of psychotherapist-related variables, e.g., therapist attachment-related behaviors. Tools that make this possible include, for example, Therapist Attunement Scales (TASc) (91) and the Patient Attachment Coding System (PACS) (92) which are used to analyze therapists’ attunement and attachment status during the psychotherapy session. By coding transcribed therapy, TASc and PACS allow it be possible to reliably determine therapist attachment style by measuring moment-by-moment discursive relational behavior within a single psychotherapy session. Research using these tools has shown that clear differences in the way the therapist functions and communicates with the patient can be observed in therapy (93).

The above tools (AAI, PACS, TAS) would significantly enrich the research considering online psychotherapy, which should be pursued in the future. However, these are tools that require more time and training for administration, transcription, and coding, thus limiting the feasibility of use in many settings; hence, they were not used in the short research presented in this article.

Due to the limitations, there is an obvious need to be careful while interpreting the results and a need to design broader tests for future research that will encompass all of the aforementioned methodological elements. The authors hope that the present research will lend itself to the creation of channeled research hypotheses and the modeling of tightly controlled research. At the same time, it is important to note the current article presents but a part of the results, with the remaining elements and data to be presented in a separate publication.

## Conclusion

The present study showed that online group psychotherapy can be effective in reducing the attachment’s dimensions of anxiety and avoidance and increasing self-esteem. No correlation has been obtained between any kind of pre-treatment attachment dimensions and psychotherapy outcomes. The study does not lend support to the online group psychotherapy as a protective factor for reducing loneliness. However, it follows that these aspects require further research, taking into account larger sample size, and the characteristics of psychotherapists’ attachment.

## Data availability statement

The data has been submitted to the Repository of the Jagiellonian University and is available under DOI number: http://dx.doi.org/10.26106/8b6s-5107.

## Ethics statement

The studies involving human participants were reviewed and approved by the Bioethics Committee of the Jagiellonian University in Krakow (1072.6120.44.2021 of the 17th of March 2021). The patients/participants provided their written informed consent to participate in this study.

## Author contributions

ZW: conceptualization, project administration, funding acquisition, investigation, data curation, formal analysis and methodology, writing—original draft, and writing—review. AK-G: project administration, funding acquisition, and data curation. SL: data curation, formal analysis and methodology, and writing—review. KS-W: data curation, validation, writing—original draft, and writing—review. BI: project administration, validation, and supervision. All authors read and approved the final manuscript.

## Conflict of interest

The authors declare that the research was conducted in the absence of any commercial or financial relationships that could be construed as a potential conflict of interest.

## Publisher’s note

All claims expressed in this article are solely those of the authors and do not necessarily represent those of their affiliated organizations, or those of the publisher, the editors and the reviewers. Any product that may be evaluated in this article, or claim that may be made by its manufacturer, is not guaranteed or endorsed by the publisher.

## References

[1] Karing C. Prevalence and predictors of anxiety, depression and stress among university students during the period of the first lockdown in Germany. *J Affect Disord Rep.* (2021) 5:100174. 10.1016/J.JADR.2021.100174 34642682 PMC8497174

[2] Liu CH Zhang E Wong GTF Hyun S Hahm H. “Chris.” factors associated with depression, anxiety, and PTSD symptomatology during the COVID-19 pandemic: clinical implications for U.S. young adult mental health. *Psychiatry Res.* (2020) 290:113172. 10.1016/J.PSYCHRES.2020.113172 32512357 PMC7263263

[3] Brooks SK Webster RK Smith LE Woodland L Wessely S Greenberg N The psychological impact of quarantine and how to reduce it: rapid review of the evidence. *Lancet.* (2020) 395:912–20. 10.1016/S0140-6736(20)30460-8/ATTACHMENT/7E45AEBB-213A-498A-8387-9AAF5FA544F1/MMC1.PDF 32112714 PMC7158942

[4] Hong JS Sheriff R Smith K Tomlinson A Saad F Smith T Impact of COVID-19 on telepsychiatry at the service and individual patient level across two UK NHS mental health Trusts. *Evid Based Ment Health.* (2021) 24:161–6. 10.1136/EBMENTAL-2021-300287 34583940 PMC8483920

[5] Feijt M de Kort Y Bongers I Bierbooms J Westerink J Ijsselsteijn W. Mental health care goes online: practitioners’ experiences of providing mental health care during the COVID-19 pandemic. *Cyberpsychol Behav Soc Netw.* (2020) 23:860–4. 10.1089/CYBER.2020.0370/ASSET/IMAGES/LARGE/CYBER.2020.0370_FIGURE2.JPEG 32815742

[6] American Psychological Association. *Psychologists Embrace Telehealth to Prevent the Spread of COVID-19.* (Vol. 21). Washington, DC: American Psychological Association (2020). p. 1–9.

[7] Varker T Brand RM Ward J Terhaag S Phelps A. Efficacy of synchronous telepsychology interventions for people with anxiety, depression, posttraumatic stress disorder, and adjustment disorder: a rapid evidence assessment. *Psychol Serv.* (2018) 16:621–35. 10.1037/ser0000239 29809025

[8] Weinberg H Rolnick A. Internet-delivered interventions for individuals, groups, families, and organizations. In: Weinberg H Rolnick A editors. *Theory and Practice of Online Therapy*. 1th ed. (Oxfordshire: Routledge) (2019). p. 1–279. 10.4324/9781315545530

[9] Weinberg H Rolnick A. Introduction. In: Weinberg H Rolnick A editors. *Theory and Practice of Online Therapy: Internet-Delivered Interventions for Individuals, Groups, Families, and Organizations.* (Oxfordshire: Routledge) (2019). p. 1–10. 10.4324/9781315545530-1

[10] Dreikurs R. Group psychotherapy and the third revolution in psychiatry. *Int J Soc Psychiatry.* (1955) 1:23–32. 10.1177/002076405500100302

[11] Yalom I Leszcz M. *THE THEORY AND PRACTICE OF GROUP PSYCHOTHERAPY.* 6th ed. New York, NY: Basic Books (2020). 818 p. 10.1111/j.1749-6632.1948.tb30971.x

[12] Bernard H Burlingame G Flores P Greene L Joyce A Kobos JC Clinical practice guidelines for group psychotherapy. *Int J Group Psychother.* (2008) 58:455–542. 10.1521/ijgp.2008.58.4.455 18837662

[13] Weinberg H. Online group psychotherapy: challenges and possibilities during COVID-19-A practice review. *Group Dyn.* (2020) 24:201–11. 10.1037/GDN0000140

[14] Kemper KJ Yun J. Group online mindfulness training: proof of concept. *J Evid Based Complement Altern Med.* (2015) 20:73–5. 10.1177/2156587214553306 25305208

[15] Dear BF Zou JB Ali S Lorian CN Johnston L Sheehan J Clinical and cost-effectiveness of therapist-guided internet-delivered cognitive behavior therapy for older adults with symptoms of anxiety: a randomized controlled trial. *Behav Ther.* (2015) 46:206–17. 10.1016/J.BETH.2014.09.007 25645169

[16] Loughnan SA Joubert AE Grierson A Andrews G Newby JM. Internet-delivered psychological interventions for clinical anxiety and depression in perinatal women: a systematic review and meta-analysis. *Arch Womens Ment Health.* (2019) 22:737–50. 10.1007/S00737-019-00961-9 31101993

[17] Breuer L Barker C. *Online Support Groups for Depression: Benefits and Barriers.* Thousand Oaks, CA: Sage Open (2015). 10.1177/2158244015574936

[18] Mahon L Leszcz M. The interpersonal model of group psychotherapy. *Int J Group Psychother.* (2017) 67:S121–30. 10.1080/00207284.2016.1218286 38449271

[19] Malat J Leszcz M. Group psychotherapy. In: Tasman A Jerald K Lieberman JA First MB Riba MB editors. *Psychiatry.* (Chichester, UK: John Wiley & Sons, Ltd) (2015). p. 1923–42. 10.1002/9781118753378.CH95

[20] Lemma A Fonagy P. Feasibility study of a psychodynamic online group intervention for depression. *Psychoanal Psychol.* (2013) 30:367–80. 10.1037/A0033239

[21] Banbury A Nancarrow S Dart J Gray L Parkinson L. Telehealth interventions delivering home-based support group videoconferencing: systematic review. *J Med Internet Res.* (2018) 20:e25. 10.2196/JMIR.8090 29396387 PMC5816261

[22] Gentry M Lapid M Clark M Rummans T. Evidence for telehealth group-based treatment: a systematic review. *J Telemed Telecare.* (2019) 25:327–42. 10.1177/1357633X18775855 29788807

[23] Marmarosh CL Forsyth DR Strauss B Burlingame GM. The psychology of the COVID-19 pandemic: a group-level perspective. *Group Dyn.* (2020) 24:122–38. 10.1037/GDN0000142

[24] Holt-Lunstad J Smith T Baker M Harris T Stephenson D. Loneliness and social isolation as risk factors for mortality: a meta-analytic review. *Perspect Psychol Sci.* (2015) 10:227–37. 10.1177/1745691614568352 25910392

[25] Forsyth DR. *Group Dynamics.* 7th ed. Boston: Cengage (2019). 10.1080/1535685X.2000.11015605

[26] Bowlby J. *Attachment and Loss.* 2nd ed. New York, NY: Basic Books (1969). 436 p.

[27] Brennan KA Clark CL Shaver PR. Self-report measurement of adult attachment: an integrative overview. In: Simpson JA Rholes WS editors. *Attachment Theory and Close Relationships.* (New York, NY: The Guilford Press) (1998). p. 46–76.

[28] Mikulincer M Shaver PR. *Attachment in Adulthood: Structure, Dynamics, and Change.* 2nd ed. New York, NY: Guilford Publications (2016). 690 p.

[29] Hazan C Shaver P. Romantic love conceptualized as an attachment process. *J Pers Soc Psychol.* (1987) 52:511–24. 10.1037/0022-3514.52.3.511 3572722

[30] Mikulincer M Shaver PR. An attachment perspective on psychopathology. *World Psychiatry.* (2012) 11:11. 10.1016/J.WPSYC.2012.01.003 22294997 PMC3266769

[31] Marmarosh CL Markin RD Spiegel EB. *Attachment in Group Psychotherapy.* Washington, DC: American Psychological Association (2013). 10.1037/14186-000

[32] Stovall-McClough KC Dozier M. Attachment states of mind and psychopathology in adulthood. In: CASSIDY J SHAVER PR editors. *Handbook of Attachment.* (New York, NY: The Guilford Press) (2016). p. 1309–52.

[33] Diener MJ Monroe JM. The relationship between adult attachment style and therapeutic alliance in individual psychotherapy: a meta-analytic review. *Psychotherapy.* (2011) 48:237–48. 10.1037/a0022425 21604902

[34] Ringel S. Attachment in Psychotherapy. *Psychoanal Soc Work.* (2008) 15:79–81. 10.1080/15228870802111856

[35] Diamond G Russon J Levy S. Attachment-based family therapy: a review of the empirical support. *Fam Process.* (2016) 55:595–610. 10.1111/FAMP.12241 27541199

[36] Crittenden PMK Dallos R. All in the family: integrating attachment and family systems theories. *Clin Child Psychol Psychiatry.* (2009) 14:389–409. 10.1177/1359104509104048 19515755

[37] Marmarosh CL. Empirical research on attachment in group psychotherapy: moving the field forward. *Psychotherapy.* (2014) 51:88–92. 10.1037/A0032523 24059737

[38] Marmarosh CL. Attachment in group psychotherapy: bridging theories, research, and clinical techniques. *Int J Group Psychother.* (2017) 67:157–60. 10.1080/00207284.2016.1267573 38449241

[39] Tasca GA Balfour L Ritchie K Bissada H. The relationship between attachment scales and group therapy alliance growth differs by treatment type for women with binge-eating disorder. *Group Dyn.* (2007) 11:1–14. 10.1037/1089-2699.11.1.1

[40] Kirchmann H Mestel R Schreiber-Willnow K Mattke D Seidler KP Daudert E Associations among attachment characteristics, patients’ assessment of therapeutic factors, and treatment outcome following inpatient psychodynamic group psychotherapy. *Psychother Res.* (2009) 19:234–48. 10.1080/10503300902798367 19396654

[41] Tasca GA Ritchie K Conrad G Balfour L Gayton J Lybanon V Attachment scales predict outcome in a randomized controlled trial of two group therapies for binge eating disorder: an aptitude by treatment interaction. *Psychother Res.* (2006) 16:106–21. 10.1080/10503300500090928

[42] Tasca GA Balfour L Ritchie K Bissada H. Developmental changes in group climate in two types of group therapy for binge-eating disorder: a growth curve analysis. *Psychother Res.* (2006) 16:499–514. 10.1080/10503300600593359

[43] Marmarosh CL Tasca GA. Adult attachment anxiety: using group therapy to promote change. *J Clin Psychol.* (2013) 69:1172–82. 10.1002/jclp.22044 24151103

[44] Rom E Mikulincer M. Attachment theory and group processes: the association between attachment style and group-related representations, goals, memories, and functioning. *J Pers Soc Psychol.* (2003) 84:1220–35. 10.1037/0022-3514.84.6.1220 12793586

[45] Flores PJ. Group psychotherapy and neuro-plasticity: an attachment theory perspective. *Int J Group Psychother.* (2010) 60:547–70. 10.1521/ijgp.2010.60.4.546 21028976

[46] Fonagy P Allison E. The role of mentalizing and epistemic trust in the therapeutic relationship. *Psychotherapy.* (2014) 51:372–80. 10.1037/A0036505 24773092

[47] Fonagy P Campbell C Bateman A. Mentalizing, attachment, and epistemic trust in group therapy. *Int J Group Psychother.* (2017) 67:176–201. 10.1080/00207284.2016.1263156 38449238

[48] Fisher S Zilcha-Mano S Fonagy P. Conceptualizing epistemic trust in psychotherapy. A triadic model. *Psychother Bull.* (2022) 57:29–35.

[49] Marmarosh CL. Emphasizing the complexity of the relationship: the next decade of attachment-based psychotherapy research. *Psychotherapy.* (2015) 52:12–8. 10.1037/a0036504 25751115

[50] Mann M Hosman CMH Schaalma HP de Vries NK. Self-esteem in a broad-spectrum approach for mental health promotion. *Health Educ Res.* (2004) 19:357–72. 10.1093/HER/CYG041 15199011

[51] Zeigler-Hill V. The connections between self-esteem and psychopathology. *J Contemp Psychother.* (2010) 41:157–64. 10.1007/S10879-010-9167-8

[52] Burlingame GM Strauss B Joyce AS. Change mechanisms and effectiveness of small group treatments. In: Lambert M editor. *Bergin and Garfield’s Handbook of Psychotherapy and Behavior Change.* (New York, NY: Wiley) (2013). p. 640–90.

[53] Fraley RC Waller NG Brennan KA. An item response theory analysis of self-report measures of adult attachment. *J Pers Soc Psychol.* (2000) 78:350–65. 10.1037/0022-3514.78.2.350 10707340

[54] Lubiewska K Głogowska K Mickiewicz K Wyrzykowska E Wiśniewski C Izdebski P. Skala experience in close relationships-revised: struktura, rzetelność oraz skrócona wersja skali w polskiej próbie. *Psychol Rozwojowa.* (2016) 2016:49–63. 10.4467/20843879PR.16.004.4793

[55] Hardt J. The symptom checklist-27-plus (SCL-27-plus): a modern conceptualization of a traditional screening instrument. *GMS Psycho Soc Med.* (2008) 5:Doc08. PMC2736518 19742283

[56] Kuncewicz D Dragan M Hardt J. Walidacja polskiej wersji kwestionariusza the symptom checklist-27-plus validation of the polish version of the symptom checklist-27-plus questionnaire. *Psychiatr Pol.* (2014) 48:345–58. 25016771

[57] Rosenberg M. *Society and the Adolescent Self-Image.* Middletown, CT: Wesleyan University Press (2015). p. 1–326. 10.2307/2575639

[58] Dzwnokowska I Lachowicz-Tabaczek K Łaguna M. *Samoocena i jej pomiar: polska adaptacja skali SES M. Rosenberga. Podrêcznik.* Warszawa: Pracownia Testoìw Psychologicznych (2008).

[59] Dzwonkowska I Lachowicz-Tabaczek K Łaguna M. Skala samooceny SES morrisa rosenberga – polska adaptacja metody. *Psychol Społeczna.* (2007) 2:164–76.

[60] Gierveld JDJ van Tilburg T. The De Jong Gierveld short scales for emotional and social loneliness: tested on data from 7 countries in the UN generations and gender surveys. *Eur J Ageing.* (2010) 7:121–30. 10.1007/S10433-010-0144-6 20730083 PMC2921057

[61] Grygiel P Humenny G Rebisz S Kwitaj P Sikorska J. Validating the polish adaptation of the 11-item de Jong Gierveld loneliness scale. *Eur J Psychol Assess.* (2013) 29:129–39. 10.1027/1015-5759/A000130

[62] Wise EA. Methods for analyzing psychotherapy outcomes: a review of clinical significance, reliable change, and recommendations for future directions. *J Pers Assess.* (2010) 82:50–9. 10.1207/S15327752JPA8201_10 14979834

[63] Temkin NR. Standard error in the Jacobson and truax reliable change index: the “classical approach” leads to poor estimates. *J Int Neuropsychol Soc.* (2004) 10:899–901. 10.1017/S1355617704106115 15637781

[64] Sauer EM Rice KG Roberts KE Richardson CME. Client attachment and change in mental health during psychotherapy. *Psychotherapy.* (2020) 57:574–9. 10.1037/pst0000304 32614199

[65] Maxwell H Tasca GA Grenon R Faye M Ritchie K Bissada H Change in attachment dimensions in women with binge-eating disorder following group psychodynamic interpersonal psychotherapy. *Psychother Res.* (2018) 28:887–901. 10.1080/10503307.2017.1278804 28128017

[66] Maxwell H Tasca GA Ritchie K Balfour L Bissada H. Change in attachment insecurity is related to improved outcomes 1-year post group therapy in women with binge eating disorder. *Psychotherapy.* (2014) 51:57–65. 10.1037/a0031100 23398032

[67] Skentzos M Naeli A Hronis A. The influence of attachment style on interpersonal learning in substance use psychotherapy groups. *Discover Psychology 2022 2:1* (2022) 2:1–11. 10.1007/S44202-021-00016-0 PMC8749339 40477784

[68] Peter B Böbel E. Significant differences in personality styles of securely and insecurely attached psychotherapists: data, reflections and implications. *Front Psychol.* (2020) 11:611. 10.3389/FPSYG.2020.00611/BIBTEX 32373012 PMC7186447

[69] Barkowski S Schwartze D Strauss B Burlingame GM Rosendahl J. Efficacy of group psychotherapy for anxiety disorders: a systematic review and meta-analysis. *Psychother Res.* (2020) 30:965–82. 10.1080/10503307.2020.1729440 32093586

[70] Barkowski S Schwartze D Strauss B Burlingame GM Barth J Rosendahl J. Efficacy of group psychotherapy for social anxiety disorder: a meta-analysis of randomized-controlled trials. *J Anxiety Disord.* (2016) 39:44–64. 10.1016/j.janxdis.2016.02.005 26953823

[71] Janis RA Burlingame GM Svien H Jensen J Lundgreen R. Group therapy for mood disorders: a meta-analysis. *Psychother Res.* (2020) 31:369–85. 10.1080/10503307.2020.1817603 32930060

[72] Puspitasari AJ Heredia D Gentry M Sawchuk C Theobald B Moore W Rapid adoption and implementation of telehealth group psychotherapy during COVID 19: practical strategies and recommendations. *Cogn Behav Pract.* (2021) 28:492–506. 10.1016/j.cbpra.2021.05.002 34188434 PMC8223010

[73] Uchino BN. Social support and health: a review of physiological processes potentially underlying links to disease outcomes. *J Behav Med.* (2006) 29:377–87. 10.1007/s10865-006-9056-5 16758315

[74] Russel GI. *Screen Relations: The Limits of Computer-Mediated Psychoanalysis and Psychotherapy.* New York, NY: Routledge (2015).

[75] Kahlon MK Aksan N Aubrey R Clark N Cowley-Morillo M Jacobs EA Effect of layperson-delivered, empathy-focused program of telephone calls on loneliness, depression, and anxiety among adults during the COVID-19 pandemic: a randomized clinical trial. *JAMA Psychiatry.* (2021) 78:616–22. 10.1001/JAMAPSYCHIATRY.2021.0113 33620417 PMC7903319

[76] Megalakaki O Kokou-Kpolou CK. Effects of biopsychosocial factors on the association between loneliness and mental health risks during the COVID-19 lockdown. *Curr Psychol.* (2021):1–12. 10.1007/s12144-021-02246-w 34456535 PMC8380098

[77] Grah M Restek-Petroviü B Keziü S Jelaviü S Lukaþiü T. Changes in the long-term psychodynamic group psychotherapy in family members of persons with psychotic disorders. *Psychiatr Danub.* (2019) 31:185–9. 31158120

[78] Kegel AF Flückiger C. Predicting psychotherapy dropouts: a multilevel approach. *Clin Psychol Psychother.* (2015) 22:377–86. 10.1002/CPP.1899 24782040

[79] Zeigler-Hill V. The interpersonal nature of self-esteem: do different measures of self-esteem possess similar interpersonal content? *J Res Pers.* (2010) 44:22–30. 10.1016/J.JRP.2009.09.005

[80] Gallagher ME Tasca GA Ritchie K Balfour L Maxwell H Bissada H. Interpersonal learning is associated with improved self-esteem in group psychotherapy for women with binge eating disorder. *Psychotherapy.* (2014) 51:66–77. 10.1037/A0031098 23398038

[81] Romeo A Tesio V Castelnuovo G Castelli L. Attachment Style and chronic pain: toward an interpersonal model of pain. *Front Psychol* (2017) 8:284. 10.3389/FPSYG.2017.00284 28286493 PMC5323382

[82] Waldinger RJ Schulz MS Barsky AJ Ahern DK. Mapping the road from childhood trauma to adult somatization: the role of attachment. *Psychosom Med.* (2006) 68:129–35. 10.1097/01.PSY.0000195834.37094.A4 16449423

[83] Fonagy P Luyten P. A developmental, mentalization-based approach to the understanding and treatment of borderline personality disorder. *Dev Psychopathol.* (2009) 21:1355–81. 10.1017/S0954579409990198 19825272

[84] Riem MME Doedée ENEM Broekhuizen-Dijksman SC Beijer E. Attachment and medically unexplained somatic symptoms: the role of mentalization. *Psychiatry Res.* (2018) 268:108–13. 10.1016/j.psychres.2018.06.056 30015108

[85] Fosha D. *Transforming Power Of Affect: A Model For Accelerated Change.* New York, NY: Basic Books (2000).

[86] Mikulincer M Shaver PR Berant E. An attachment perspective on therapeutic processes and outcomes. *J Pers.* (2013) 81:606–16. 10.1111/J.1467-6494.2012.00806.X 22812642

[87] Illing V Tasca GA Balfour L Bissada H. Attachment dimensions and group climate growth in a sample of women seeking treatment for eating disorders. *Psychiatry.* (2011) 74:255–69. 10.1521/psyc.2011.74.3.255 21916631

[88] Dozier M Kobak RR. Psychophysiology in attachment interviews: converging evidence for deactivating strategies. *Child Dev.* (1992) 63:1473. 10.2307/1131569 1446563

[89] Levy KN Johnson BN. Attachment and psychotherapy: implications from empirical research. *Can Psychol.* (2019) 60:178–93. 10.1037/CAP0000162

[90] Ravitz P Maunder R Hunter J Sthankiya B Lancee W. Adult attachment measures: a 25-year review. *J Psychosom Res.* (2010) 69:419–32. 10.1016/J.JPSYCHORES.2009.08.006 20846544

[91] Talia A Muzi L Lingiardi V Taubner S. How to be a secure base: therapists’ attachment representations and their link to attunement in psychotherapy. *Attach Hum Dev.* (2020) 22:189–206. 10.1080/14616734.2018.1534247 30336734

[92] Talia A Miller-Bottome M Daniel SIF. Assessing attachment in psychotherapy: validation of the patient attachment coding system (PACS). *Clin Psychol Psychother.* (2017) 24:149–61. 10.1002/cpp.1990 26596847

[93] Talia A Taubner S Miller-Bottome M. Advances in research on attachment-related psychotherapy processes: seven teaching points for trainees and supervisors. *Res Psychother.* (2019) 22:359–68. 10.4081/RIPPPO.2019.405 32913812 PMC7451314

